# Efficacy and safety of PEG-IFN α-2b and tenofovir amibufenamide in combination therapy for chronic hepatitis B

**DOI:** 10.1186/s12879-025-12296-1

**Published:** 2025-12-19

**Authors:** Wen Zhao, Longcan Li, Shihui Liu, Wenjie Zhang, Yufeng Gao, Zonghao Zhao, Xiaojun Liu, Yi Luo, Dongdong Li, Chuanmiao Liu

**Affiliations:** 1 Department of Infectious Diseases, The First Affiliated Hospital of Bengbu Medical University, No. 287 Changhuai Road, Bengbu, Anhui Province 233000 China; 2 Core Cooperative Unit of National Clinical Research Center for Infectious Diseases, Bengbu, 233000 China; 3 Key Laboratory of Infection and Immunity of Anhui province, Bengbu, 233000 China; 4 Department of Infectious Diseases, The First Affiliated Hospital of Anhui Medical University, Hefei, 230000 China; 5 Department of Infectious Diseases, The First Affiliated Hospital of University of Science and Technology of China, Hefei, 230001 China

**Keywords:** Chronic hepatitis B, Combination therapy, HBsAg clearance, PEG-IFN α-2b, Tenofovir amibufenamide

## Abstract

**Background:**

This study aimed to explore the efficacy and safety of combination therapy with PEG-IFN α-2b and tenofovir amibufenamide (TMF) for the treatment of chronic hepatitis B (CHB).

**Methods:**

This multicenter study enrolled 84 CHB patients, who received PEG-IFN α-2b (180 µg/weekly) and TMF (25 mg/day) for 48 weeks. Clinical and laboratory assessments were performed at baseline and at 12-week intervals (weeks 12, 24, 36, and 48). Serologic response (SR) was defined as hepatitis B surface antigen (HBsAg) loss (< 0.05 IU/mL), with or without HBsAg seroconversion (HBsAg < 0.05 IU/mL and HBsAb > 10 mIU/mL). Adverse events (AEs) were monitored at each assessment. Logistic regression and receiver operating characteristic curve analyses were used to identify predictors of HBsAg clearance.

**Results:**

The combination therapy of PEG-IFN α-2b and TMF resulted in significant reductions in HBsAg levels from baseline at weeks 24, 36, and 48. At these time points, the proportion of patients with undetectable HBV DNA increased progressively, with the proportion reaching 94.7% at week 48. In the SR group, the baseline HBsAg and HBeAg levels were significantly lower than those in the non-serological response (NSR) group, with greater reductions in HBsAg observed at weeks 12 and 24. Multivariate analysis revealed that baseline HBsAg levels and the degree of HBsAg decline at week 24 were independent predictors of HBsAg loss, with odds ratios of 4.609 and 3.237, respectively. The diagnostic performance of baseline HBsAg levels and their decline at week 24 demonstrated areas under the curves (AUCs) of 0.856 and 0.821, respectively, with a combined AUC of 0.908. The cumulative HBsAg clearance rates were 25% in treatment-naïve patients and 26.7% in treatment-experienced patients. The most frequently reported AEs included fever, fatigue, rash, alopecia, elevated ALT and AST levels, neutropenia, and thrombocytopenia.

**Conclusions:**

PEG-IFN α-2b and TMF combination therapy effectively reduced HBsAg levels in CHB patients. Baseline HBsAg levels and the magnitude of their decline by week 24 served as robust predictors of serological response, exhibiting high diagnostic value.

## Highlights

First evaluation of the PEG-IFN α-2b and Tenofovir Alafenamide (TAF) combination for chronic hepatitis B (CHB).

The combination regimen demonstrates a significant rate of HBsAg loss.

Baseline HBsAg levels and on-treatment HBsAg reduction at week 24 are strong predictors of treatment success.

Provides a new strategy for optimizing functional cure in CHB patients.

## Background

Chronic hepatitis B virus (HBV) infection is estimated to affect approximately 254 million individuals globally, with 79.7 million of these cases in China. According to 2022 World Health Organization (WHO) surveillance, the diagnosis rate and treatment coverage for HBV in China were as low as 24.0% and 6.4%, respectively. Globally, HBV infection is responsible for approximately 1.3 million annual deaths, with China accounting for an estimated 308,000 fatalities each year due to HBV-related liver diseases [1]. HBV infection during pregnancy is associated with an elevated risk of adverse maternal and neonatal outcomes, such as gestational diabetes mellitus, premature rupture of membranes, and neonatal asphyxia [2]. Serum HBV DNA concentration has been identified as a significant risk factor for intrauterine infection [3]. Moreover, HBV represents a leading cause of fulminant hepatitis. When manifesting in late pregnancy, it may present as a distinct clinical syndrome marked by rapid progression and severe manifestations, leading to alarmingly high maternal and fetal mortality [4, 5]. To achieve a functional cure for chronic hepatitis B (CHB), both HBV DNA and hepatitis B surface antigen (HBsAg) must be undetectable. Current antiviral treatment strategies involve two classes of agents: immunomodulators such as pegylated interferon (PEG-IFN) and direct-acting antivirals, notably nucleos(t)ide analogs (NAs). Among these, entecavir (ETV), tenofovir disoproxil fumarate (TDF), and tenofovir alafenamide (TAF) are commonly used as first-line treatments because of their strong antiviral efficacy and low resistance profile [6]. While NAs are highly effective in achieving virologic suppression during treatment, their ability to maintain this response after therapy discontinuation is often limited. In contrast, PEG-IFN activates immune cells and induces the expression of interferon-stimulated genes (ISGs), which produce antiviral proteins that directly target the virus [7]. Additionally, PEG-IFN promotes the degradation of HBV pregenomic RNA (pgRNA) and core particles, and plays a role in modulating the epigenetic regulation of cccDNA, further contributing to the suppression of HBV replication [8, 9]. However, PEG-IFN treatment results in a modest reduction in serum HBsAg levels, with durable off-treatment responses observed in only a limited proportion of patients.

Compared with TAF, TMF, a novel prodrug of tenofovir developed via ProTide technology, features an additional methyl group. This modification enhances the drug’s lipid solubility, facilitating its efficient delivery to hepatocytes while minimizing systemic exposure to tenofovir [10]. Furthermore, TMF shares a metabolic pathway similar to that of TAF but has reduced stability in HBV-infected hepatocytes. This leads to more rapid activation of the drug, enhancing its anti-HBV effects [10, 11]. TMF was approved by the National Medical Products Administration in June 2021 for the antiviral treatment of CHB in China. In phase III clinical trials, TMF demonstrated comparable efficacy to TDF over 48 weeks, with similar rates of HBV DNA suppression to < 20 IU/mL and HBeAg loss. Achieving HBsAg clearance or seroconversion with TMF remains challenging, similar to the outcomes observed with other oral anti-HBV agents [12].

The pharmacokinetics of interferon therapy can be improved by incorporating polyethylene glycol into the formulation. A meta-analysis demonstrated that compared with standard interferon treatment, PEG-IFN therapy results in superior outcomes. PEG-IFN α-2a and PEG-IFN α-2b, two distinct pegylated interferon formulations, are employed in the treatment of hepatitis B. The mechanistic rationale for combining NAs and PEG-IFN involves the observation that these two classes of drugs have differential effects on innate and adaptive immunity and that viral suppression mediated by NAs directly enhances the subsequent immunologic response to PEG-IFN [13, 14].

Evidence on the combination of TMF and PEG-IFN α-2b for CHB remains limited. We enrolled 84 CHB patients receiving combined TMF and PEG-IFN α-2b, investigating this regimen’s potential to enhance functional cure rates and identify predictive biomarkers.

## Methods

### Patient recruitment

This multicenter, retrospective, real-world clinical study was conducted at the Infectious Diseases Department of the First Affiliated Hospital of Bengbu Medical University, the First Affiliated Hospital of Anhui Medical University, and Anhui Provincial Hospital from February 2024 to January 2025. A total of 84 CHB patients were enrolled during this period. CHB diagnosis was confirmed in accordance with the Guidelines for the Prevention and Treatment for Chronic Hepatitis B (2022 Version) [15]. The enrolled patients received subcutaneous injections of PEG-IFN α-2b (180 µg; Pegbin, Xiamen Amoytop Biotech Co., Ltd., Xiamen, China) once weekly and oral TMF (25 mg; Hengmu, Hansoh Pharma, Lianyungang, China) once daily for 48 weeks. Routine clinical and laboratory assessments were performed at baseline and every 12 weeks (week 0, week 12, week 24, week 36 and week 48) during PEG-IFN treatment. Serologic response was defined as the loss of HBsAg (< 0.05 IU/mL), with or without HBsAg seroconversion (defined as HBsAg < 0.05 IU/mL and HBsAb >10 mIU/mL). Patients were classified into two groups: treatment-experienced and treatment-naïve. The treatment-experienced patients were those who had received NAs (ETV, TDF or TAF) for more than 24 weeks. Treatment- naïve patients had never received any antiviral therapy, including both interferon and NAs.

The exclusion criteria were as follows: (1) allergy to PEG-IFN α-2b or its components; (2) decompensated cirrhosis or liver cancer; (3) concurrent hepatitis C, hepatitis D, hepatitis E, alcoholic liver disease, autoimmune liver disease, primary biliary cirrhosis, or other conditions causing liver damage; (4) pregnancy or plans to become pregnant in the near future; and (5) a history of severe cardiovascular disease, serious infections, mental disorders, retinal diseases, autoimmune diseases, uncontrolled diabetes, thyroid disorders, retinopathy, or other significant comorbidities.

This study was approved by the Ethics Committee of the First Affiliated Hospital of Bengbu Medical University (approval number: [2023]KY044) and was conducted in accordance with the Declaration of Helsinki. Written informed consent was obtained from all patients prior to their inclusion in the study.

### Laboratory measurements

Blood samples were collected at each visit. Routine blood tests were performed using an automated hematology analyzer (Beckman Coulter LH750, CA, USA). Biochemical parameters, including alanine aminotransferase (ALT), aspartate aminotransferase (AST) and total bilirubin (TBil) were measured with an automatic biochemistry analyzer (Olympus AU2700; Olympus, Tokyo, Japan). Thyroid function tests were also conducted via an automatic biochemistry analyzer (ADVIA Centaur XPT; SIEMENS, Germany). Serum HBV DNA levels were quantified via real-time fluorescence quantitative polymerase chain reaction using a commercial HBV DNA detection kit (Xiamen Amplly, Xiamen, Fujian Province, China), with a detection limit of 50 IU/ml. The levels of HBsAg (LOD < 0.05 IU/mL), HBsAb, HBeAg (LOD < 1.0 signal/cutoff, S/CO), HBeAb and HBcAb were measured via a commercial chemiluminescent microparticle immunoassay kit with the Alinity i system (Abbott Laboratories, USA).

### Adverse events

The analysis of adverse events (AEs) revealed a range of potential side effects, including but not limited to fever, fatigue, rash, alopecia, insomnia, ALT and AST elevation, neutropenia, thrombocytopenia, anemia, depression, and anxiety. All AEs were assessed and graded according to the Common Terminology Criteria for Adverse Events (CTCAE, Version 5.0) to standardize the evaluation of their severity.

### Statistical analysis

HBsAg and HBeAg concentrations were log_10_-transformed for analysis. Statistical analyses and graphing were performed via SPSS version 26.0 (SPSS Inc., USA) and GraphPad Prism version 9.0 (GraphPad Software, USA). Continuous variables are presented as mean ± standard error (SE) for normally distributed data or median (interquartile range, IQR) for non-normally distributed data. Categorical variables are expressed as counts (percentages). Group comparisons for continuous variables were performed using Student’s *t* tests (for two groups) or one-way ANOVA (for more than two groups) for parametric data, and the Mann-Whitney U test (for two groups) or the Kruskal-Wallis test (for more than two groups) for non-parametric data. Differences in the proportions of categorical variables were compared via the chi-square test or Fisher’s exact test. Logistic regression analysis was conducted to identify factors associated with HBsAg clearance, with odds ratios (ORs) and 95% confidence intervals (CIs) reported for each factor. A receiver operating characteristic (ROC) curve was constructed to evaluate the predictive value of the signature, and the area under the curves (AUCs) was calculated. A two-sided *P*-value < 0.05 was considered statistically significant.

## Results

### Baseline characteristics of the enrolled patients

**Fig. 1:**
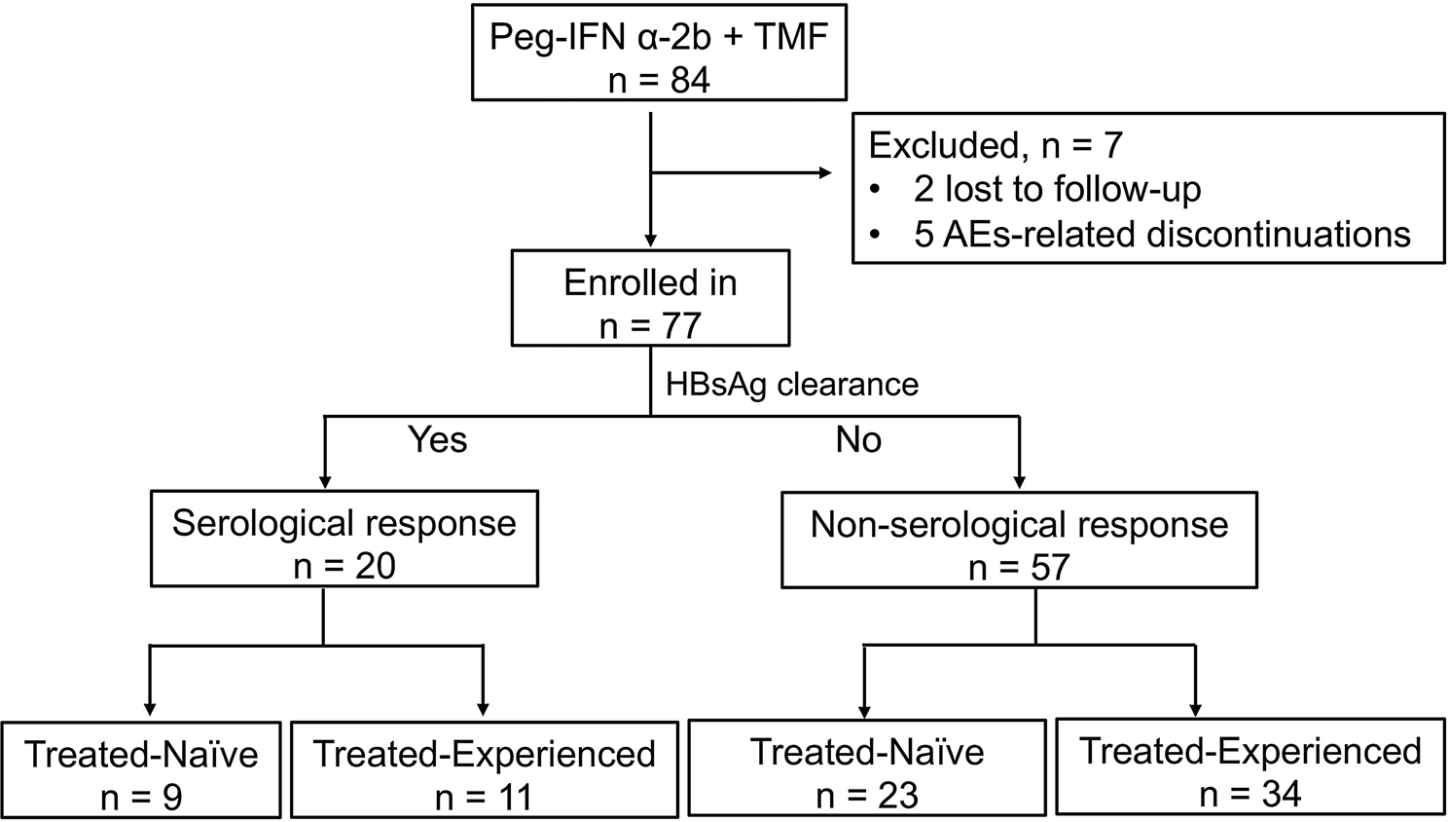
Patient enrollment flowchart. TMF, tenofovir amibufenamide; AEs, adverse events; HBsAg, hepatitis B surface antigens

A total of 84 individuals were screened for this study. Seven patients were excluded, two due to voluntary withdrawal and five due to treatment-related AEs (i.e., rash, pain, and hair loss). Ultimately, 77 patients with CHB infection who received combination therapy were included. All patients received at least 24 weeks of treatment, with 59 patients completing 48 weeks of treatment. A total of 20 patients achieved a serological response (Fig. 1). The baseline characteristics of the enrolled subjects are summarized in Table 1. Among the patients, 50 (64.9%) had baseline HBsAg levels less than 1000 IU/ml, and 22 (28.57%) had HBsAg levels less than 100 IU/ml. Clinical indices, including blood routine tests and liver function markers, were within normal reference ranges at baseline (Table 1).

**Table 1:** Baseline clinical characteristics of the enrolled patients

Variables	Total (*n* = 77)
Age (years)	38.0 (34.0, 46.0)
Male gender, n (%)	61 (79.22)
Treat history-Naïve, n (%)	32 (41.6)
HBsAg, log_10_ IU/mL	2.762 (1.700, 3.294)
HBeAg-positive, n (%)	14 (18.18)
HBeAg, log_10_ IU/mL	-0.398 (-0.434, -0.266)
HBV DNA undetectable, n (%)	42 (54.5)
WBC, 10^9^/L	6.50 ± 1.53
PLT, 10^9^/L	151.00 (135.50, 213.00)
HGB, g/L	145.86 ± 15.148
ALT, U/L	27.0 (20.7, 32.0)
AST, U/L	24.0 (20.7, 32.0)
TBil, µmol/L	11.1 (8.68, 14.7)
APRI score	0.26 (0.18, 0.38)
FIB-4 score	1.10 (0.82, 1.69)

Abbreviations: HBsAg, hepatitis B surface antigen; HBeAg, hepatitis B virus e antigen; WBC, white blood cell; PLT, platelet; HGB, hemoglobin; ALT, alanine aminotransferase; AST, aspartate aminotransferase; TBIL, total bilirubin; APRI, AST to platelet ratio index; FIB-4, fibrosis-4 index

**Fig. 2:**
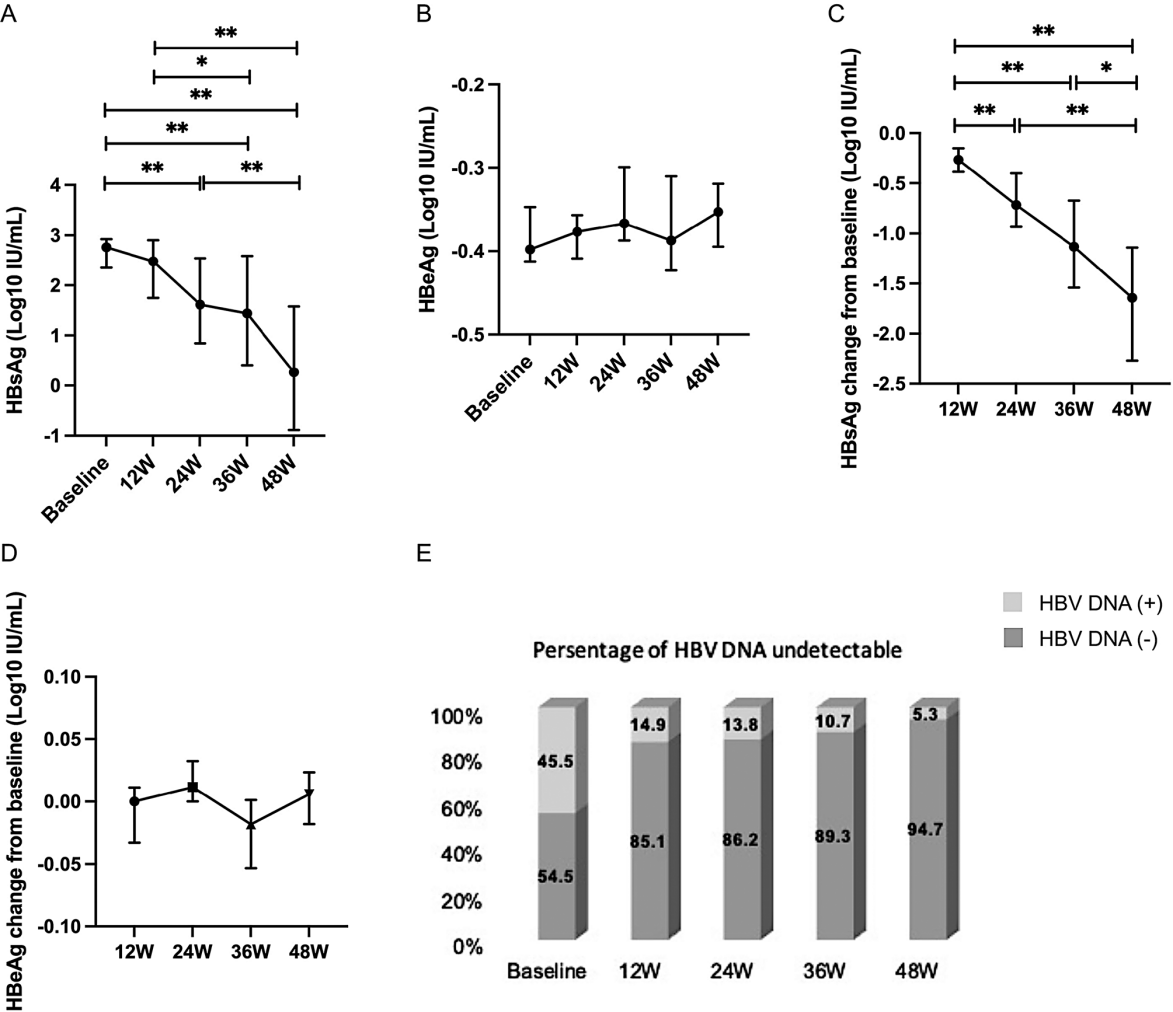
Serological response dynamics in CHB patients during PEG-IFN α-2b and TMF combination therapy. (**A**-**B**) Serum HBsAg (**A**) and HBeAg (**B**) levels at weeks 12, 24, 36, and 48 during treatment. (**C**-**D**) Changes in HBsAg (**C**) and HBeAg (**D**) from baseline to weeks 12, 24, 36, and 48. (**E**) Proportions of patients with undetectable HBV DNA at each time point. HBsAg, hepatitis B surface antigen; HBeAg, hepatitis B virus e antigen. ^*^P < 0.05, ^**^P < 0.01

Compared with baseline levels, HBsAg levels showed significant reductions of 1.61 log_10_ IU/mL, 1.44 log_10_ IU/mL, and 0.27 log_10_ IU/mL at weeks 24, 36 and 48, respectively (all *P* < 0.001). At both 36 and 48 weeks, the HBsAg levels were significantly lower than those at week 12 (2.48 log₁₀ IU/mL; *P* = 0.020 and *P* < 0.001, respectively). Additionally, a significant decrease in HBsAg was observed at week 48 compared with week 24 (*P* = 0.004; Fig. 2A). Among the 14 HBeAg-positive patients at baseline, 3 achieved HBeAg clearance by week 48. However, no significant changes in HBeAg levels were observed at any time point (all *P* > 0.05; Fig. 2B). The reduction in HBsAg from baseline was significantly greater at weeks 24 (*P* = 0.009), 36 (*P* < 0.001), and 48 (*P* < 0.001) than at week 12. The reduction at week 48 was also significantly greater than that at both week 24 (*P* < 0.001) and week 36 (*P* = 0.019; Fig. 2C). In contrast, HBeAg levels did not significantly change over time (all *P* > 0.05; Fig. 2D). The proportion of patients with undetectable HBV DNA was significantly higher at weeks 12, 24, 36, and 48 than at baseline (all *P* < 0.001; Fig. 2E).

### Characteristics of chronic HBV-infected patients who achieved HBsAg loss following PEG-IFN α-2b and TMF combination therapy during follow-up

We classified CHB patients into two groups, the serological response (SR) group and the non-serological response (NSR) group, on the basis of HBsAg loss or seroconversion during the follow-up period of combination therapy. Baseline serological, virological, and liver function indicators were compared between the two groups. Among the 20 patients in the SR group, 18 completed 48 weeks of treatment, whereas 41 of 57 in the NSR group completed 48 weeks of treatment. Among the 20 patients who achieved HBsAg loss, 16 (80%) developed anti-HBs positivity by the final follow-up. In the NSR group, by the end of treatment, HBsAg levels were < 100 IU/mL in 26/57 (45.6%) patients and < 10 IU/ mL in 14/57 (24.6%) patients. Baseline HBsAg levels and HBeAg positivity rates were significantly lower in the SR group than in the NSR group (all *P* < 0.001). In the SR group, significant declines in HBsAg were observed at both 12 weeks (*P* = 0.001) and 24 weeks (*P* < 0.001; Table 2).

**Table 2:** Comparison of characteristics between the serological response and non-serological response groups

Variables	SR	NSR	F/Z/X^2^	*P* value
	*n* = 20	*n* = 57		
Baseline				
Age (years)	41 (37, 48)	37 (34, 46)	1.097	0.180
Male gender, n (%)	16 (80.00)	45 (78.95)	0.010	0.920
Treat history-Naïve, n (%)	9 (45.00)	23 (40.35)	0.132	0.717
HBeAg-positive, n (%)	1 (5.00)	13 (22.81)	31.182	< 0.001
HBeAg, log_10_ IU/mL	-0.41 (-0.46, -0.35)	-0.39 (-0.43, -0.21)	-1.514	0.122
HBsAg, log_10_ IU/mL	1.65 (0.88, 2.30)	2.92 (2.33, 3.46)	2.318	< 0.001
HBV DNA undetectable, n (%)	11 (55.00)	31 (54.39)	0.636	0.425
WBC, ×10^9^/L	6.71 ± 1.37	6.41 ± 1.60	0.734	0.465
PLT, ×10^9^/L	144.00 (135.00, 151.50)	157.00 (139.00, 223.00)	-1.683	0.092
HGB, g/L	142.60 ± 10.81	146.85 ± 16.54	-1.064	0.291
ALT, U/L	26.00 (19.00, 31.00)	31.00 (19.00, 44.50)	-1.087	0.277
AST, U/L	21.00 (19.00, 31.00)	25.00 (21.00, 34.00)	-1.451	0.147
TBil, µmol/L	10.60 (8.60, 14.80)	11.50 (8.60, 14.60)	-0.253	0.800
Change during treatment				
HBsAg decline at week12, log_10_ IU/mL	-0.93 (-1.97, -1.20)	-0.22 (-0.53, -0.05)	1.917	0.001
HBsAg decline at week24, log_10_ IU/mL	-2.10 (-3.17, -0.84)	-0.51 (-0.96, -0.18)	2.241	< 0.001
ALT elevation from baseline to week 12	20.00 (3.30, 61.78)	25.50 (12.75, 61.00)	-0.852	0.394

Abbreviations: SR, serological response; NSR, nonserological response; HBeAg, hepatitis B virus e antigen; HBsAg, hepatitis B surface antigens; WBC, white blood cell; PLT, platelet; HGB, hemoglobin; ALT, alanine aminotransferase; AST, aspartate aminotransferase; TBIL, total bilirubin

**Fig. 3:**
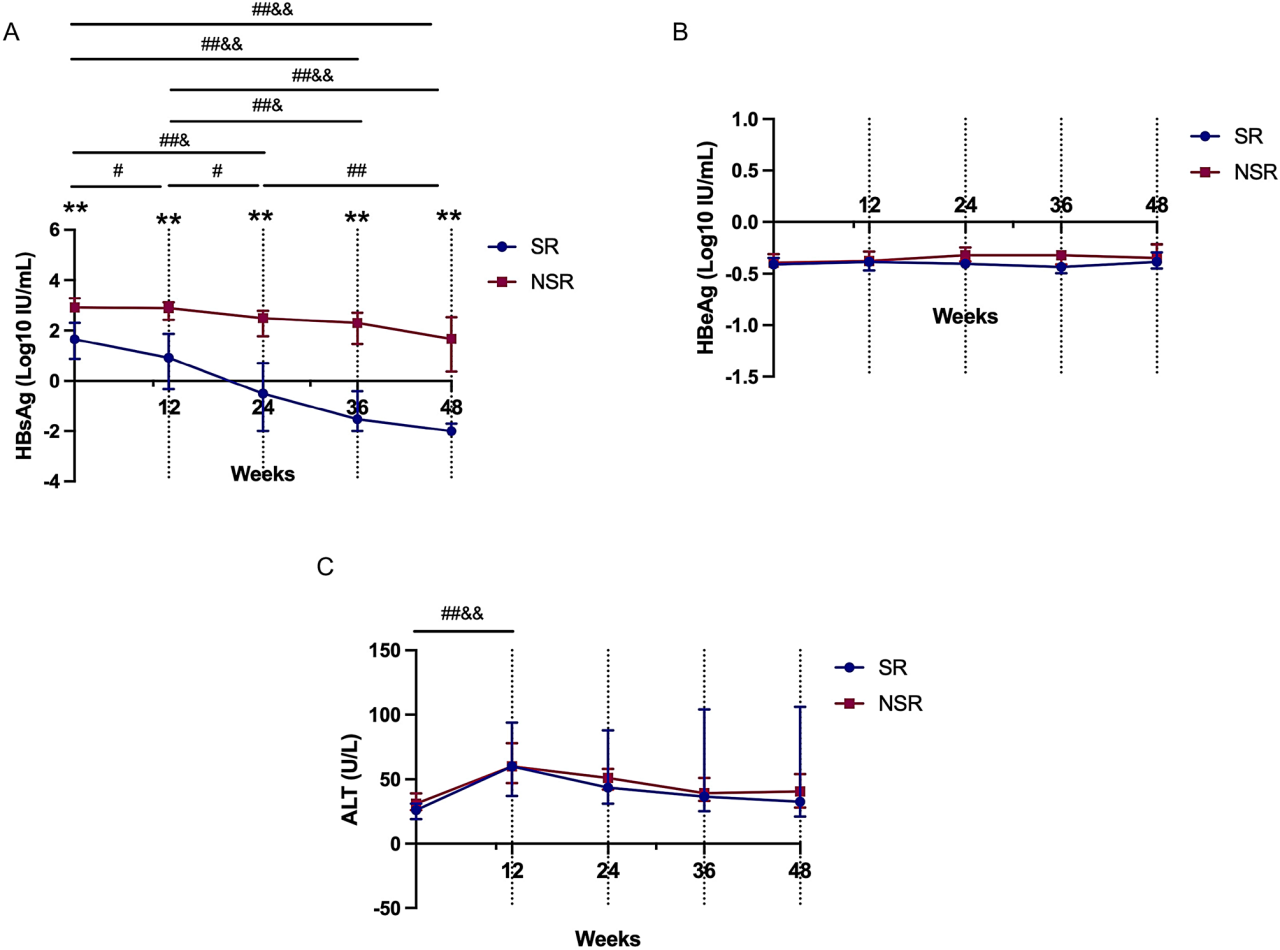
Dynamic changes in HBsAg, HBeAg and ALT levels over 48 weeks of therapy in the SR and NSR groups. Median HBsAg levels (**A**), HBeAg levels (**B**) and ALT levels (**C**) during therapy in the SR group and NSR group. SR, serological response; NSR, non-serological response; HBsAg, hepatitis B surface antigen; HBeAg, hepatitis B virus e antigen; ALT, alanine aminotransferase. Statistical significance: ^*^P < 0.05, ^**^P < 0.01 between the SR and NSR groups; ^#^*P* < 0.05, ^##^*P* < 0.01 between time points in the SR group; ^&^*P* < 0.05 and ^&&^*P* < 0.01 between time points in the NSR group

HBsAg levels were significantly lower in the SR group than in the NSR group from baseline through 48 weeks of therapy (all *P* < 0.001). In the SR group, the median HBsAg level significantly declined from 1.65 log_10_ IU/mL at baseline to 0.92 log_10_ IU/mL at week 12 (*P* = 0.020), -0.50 log_10_ IU/mL at week 24 (*P* < 0.001), -1.52 log_10_ IU/mL at week 36 (*P* < 0.001), and − 2.00 log_10_ IU/mL at week 48 (*P* < 0.001). In contrast, in the NSR group, the median HBsAg level decreased from 2.92 log_10_ IU/mL at baseline to 2.49 log_10_ IU/ mL at week 24 (*P* = 0.031), 2.31 log_10_ IU/mL at week 36 (*P* = 0.008), and 1.67 log_10_ IU/ mL at week 48 (*P* < 0.001; Fig. 3A). In both the SR and NSR groups, the median HBsAg level continued to decrease from week 12 to week 36 and week 48 (all *P* < 0.05). Serum HBeAg levels did not significantly change from baseline at any time point in either the SR or NSR group (all *P* > 0.05; Fig. 3B). Additionally, the median ALT level increased significantly to 60 U/L in both groups by week 12 (both *P* < 0.001; Fig. 3C).

### Factors associated with the serological response following PEG-IFN α-2b and TMF combination therapy

Table 3 presents an analysis of factors associated with HBsAg loss in CHB patients treated with PEG-IFN α-2b and TMF. Univariate analysis of 16 variables identified four statistically significant predictors, which were then included in the multivariate analysis. In the final multivariate model, significant independent predictors of HBsAg loss were lower baseline HBsAg levels [OR (95% CI): 4.609 (1.689–12.575), *P* = 0.003] and a greater decline in HBsAg levels from baseline to week 24 [OR (95% CI): 3.237 (1.390–7.536), *P* = 0.006].

**Table 3:** Multivariate analysis of predictive factors for HBsAg loss in CHB patients treated with PEG-IFN α-2b and TMF

Variables	β value	Wald	OR (95%CI)	*P* value
HBeAg-positive, n (%)	1.481	0.693	4.398 (0.134, 143.964)	0.405
HBsAg, log_10_ IU/mL	1.528	8.901	4.609 (1.689, 12.575)	0.003
HBsAg decline at week12, log_10_ IU/mL	0.253	0.159	1.287 (0.372, 4.459)	0.690
HBsAg decline at week24, log_10_ IU/mL	1.175	7.420	3.237 (1.390, 7.536)	0.006
Constant	-0.557	0.266	0.573	0.606

Abbreviations: HBeAg, hepatitis B virus e antigen; HBsAg, hepatitis B surface antigen; OR, odds ratio; CI, confidence interval

**Fig. 4:**
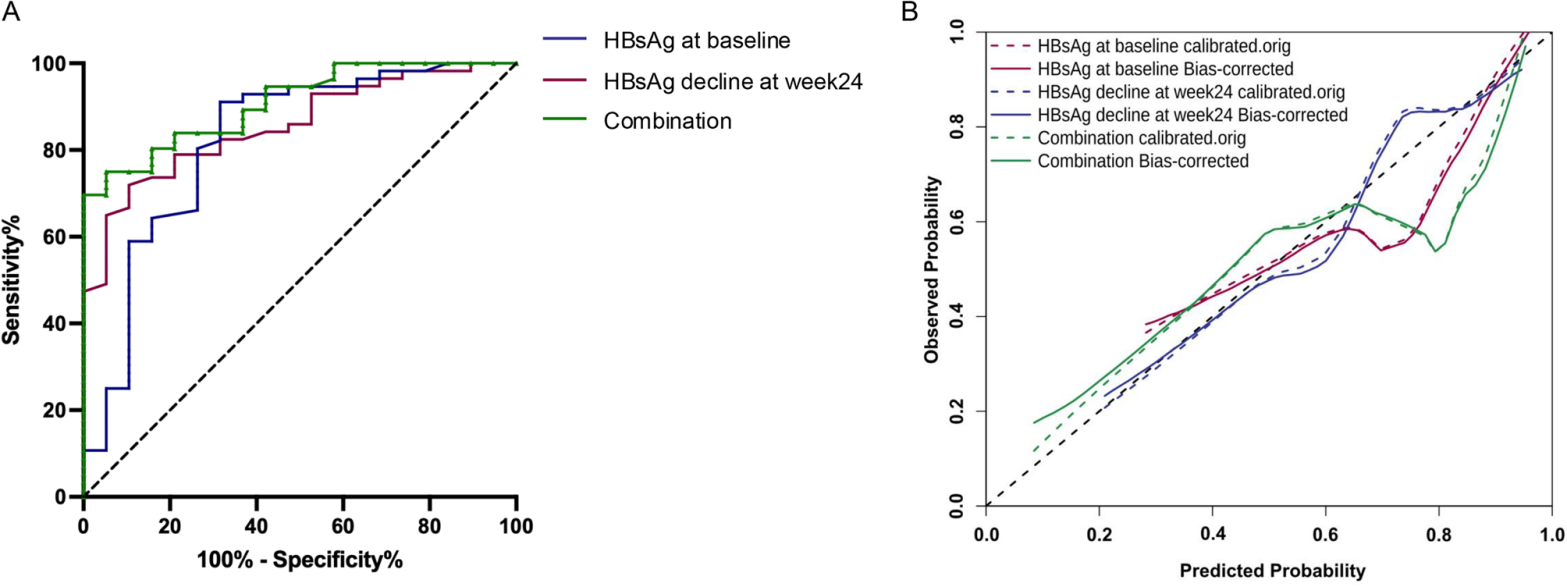
ROC and calibration curves for predicting the serological response. (**A**) ROC curves for baseline HBsAg levels, HBsAg reduction, and their combination in predicting the serological response. (**B**) Calibration curve comparing predicted versus actual probabilities for baseline HBsAg, HBsAg reduction, and the combined model. HBsAg, hepatitis B surface antigen

We evaluated the predictive performance of baseline HBsAg levels and the decline in HBsAg levels from baseline to week 24 in determining the serological response among CHB patients. The ROC curves are presented in Fig. 4. For predicting the serological response, baseline HBsAg level yielded an AUC of 0.856 (95% CI: 0.771–0.941), whereas the decrease in HBsAg level from baseline to week 24 demonstrated a comparable AUC of 0.821 (95% CI: 0.697–0.944; *P* = 0.677 vs. baseline). The combination of baseline HBsAg levels and HBsAg decline at week 24 resulted in an AUC of 0.908, which was not significantly higher than the AUC of either predictor alone (both *P* > 0.05; Table 4; Fig. 4).

**Table 4:** Diagnostic performance of baseline HBsAg levels and HBsAg decline in predicting serological response

Variables	AUC (95%CI)	Cutoff	Se%	Sp%	Youden index
Baseline HBsAg, log_10_ IU/mL	0.856 (0.771–0.941)	2.765	64.91	94.74	0.597
HBsAg decline at week24, log_10_ IU/mL	0.821 (0.697–0.944)	0.595	58.93	89.47	0.484
Combination	0.908 (0.844–0.973)	0.794	75	94.74	0.697

Abbreviations: AUC, area under the curve; Se, sensitivity; Sp, specificity; HBsAg, hepatitis B surface antigen

### Characteristics of treatment-naïve vs. treatment-experienced CHB patients receiving PEG-IFN α-2b and TMF combination therapy

**Fig. 5:**
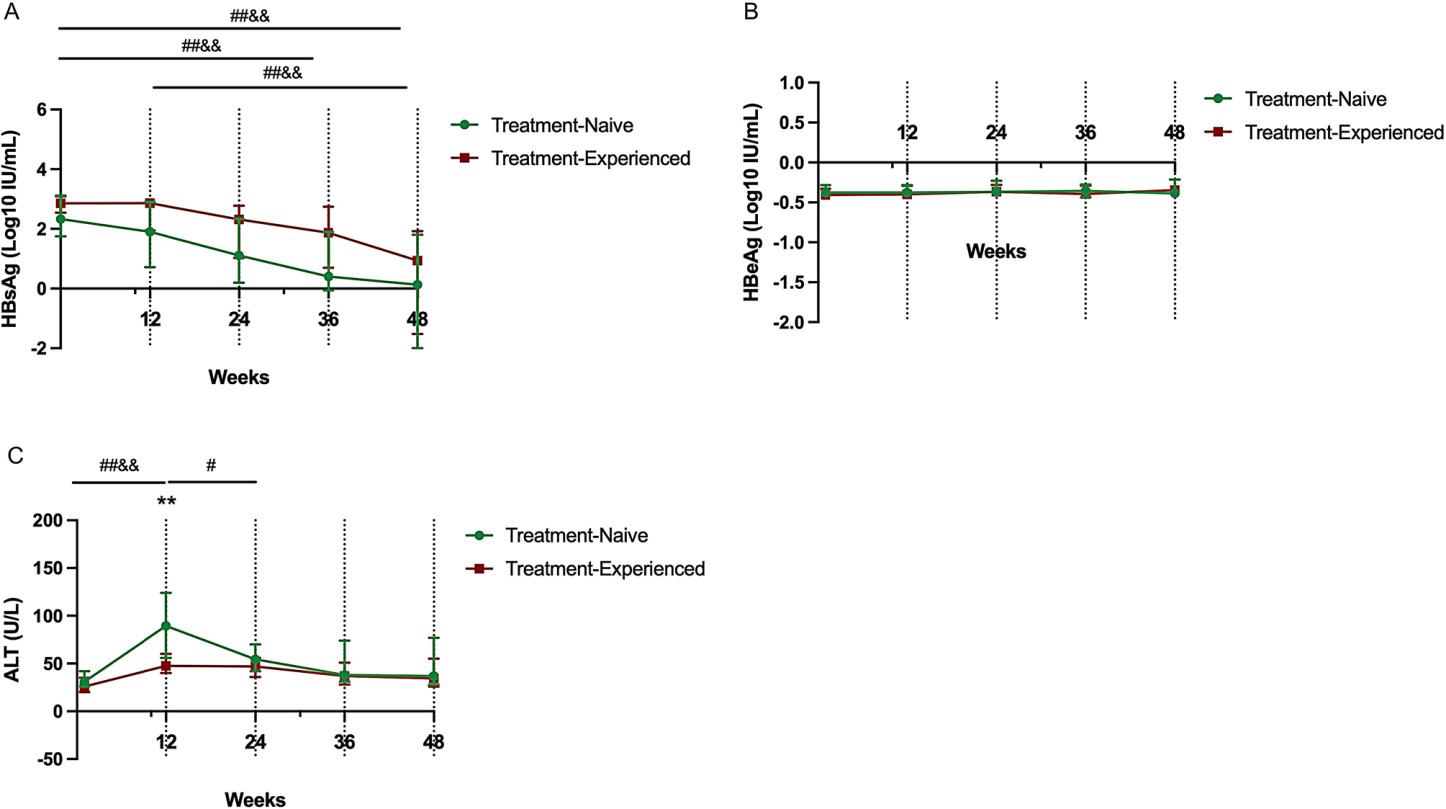
Time course of serum HBsAg, HBeAg and ALT levels during 48-weeks of therapy in the treatment-naïve and treatment-experienced groups. Median HBsAg dynamics (**A**), HBeAg dynamics (**B**), and ALT dynamics levels (**C**) over the course of therapy in the TN and TE groups. TN, treatment-naïve; TE, treatment-experienced; HBsAg, hepatitis B surface antigen; HBeAg, hepatitis B virus e antigen; ALT, alanine aminotransferase. Statistical significance: ^**^P < 0.01 between the TN and TE groups; ^#^*P* < 0.05, ^##^*P* < 0.01 between time points in the TN group; ^&&^*P* < 0.01 between time points in the TE group

Table 5 summarizes the baseline characteristics of 32 treatment-naïve (TN) and 45 treatment-experienced (TE) patients. Compared with the TE group, the TN group presented a greater male predominance (*P* < 0.001) and elevated median ALT (*P* = 0.032) and AST (*P* = 0.014) levels. In contrast, TE patients were significantly older (*P* = 0.002), more frequently HBeAg-positive (*P* < 0.001) and had a greater proportion of patients with undetectable HBV DNA (*P* < 0.001). The cumulative HBsAg clearance rates were 25% in the TN group and 26.7% in the TE group. In the TN group, the median HBsAg level significantly declined from 2.33 log_10_ IU/mL at baseline to 0.41 log_10_ IU/ mL at week 36 (*P* = 0.001), and further to 0.13 log_10_ IU/ mL at week 48 (*P* < 0.001). In the TE group, the median HBsAg level decreased from 2.85 log_10_ IU/mL at baseline to 1.87 log_10_ IU/ mL at week 36 (*P* = 0.003), and to 0.93 log_10_ IU/ mL at week 48 (*P* < 0.001). Significant declines in HBsAg levels were observed from week 12 (2.26 log_10_ IU/mL in the TN group vs. 2.74 log_10_ IU/mL in the TE group) to week 48 (*P* = 0.003 for TN and *P* < 0.001 for TE; Fig. 5A). The serum HBeAg levels did not significantly change at any time point in either group (all *P* > 0.05; Fig. 5B). The median ALT level at week 12 was significantly greater in the TN group than in the TE group (*P* = 0.002; Fig. 5C). Furthermore, the ALT level decreased to 54 U/L in the TN group by week 24 (*P* = 0.019).

**Table 5:** Characteristics of treatment-naïve patients and treatment-experienced patients

Variables	Treatment- Naïve	Treatment-Experienced	F/Z/X^2^	*P* value
	*n* = 32	*n* = 45		
Baseline				
Age (years)	36 (32, 42.5)	41.5 (35, 49)	-3.066	0.002
Male gender, n (%)	27 (84.4)	34 (75.6)	26.299	< 0.001
HBeAg-positive, n (%)	5 (15.6)	9 (20.0)	31.182	< 0.001
HBeAg, log_10_ IU/mL	-0.38 (-0.44, -0.25)	-0.41 (-0.43, -0.27)	-0.449	0.653
HBsAg, log_10_ IU/mL	2.33 (1.46, 3.22)	2.85 (2.00, 3.42)	-1.199	0.231
HBV DNA undetectable (< 50 IU/mL), n (%)	1 (3.1)	41 (91.1)	58.393	< 0.001
WBC, ×10^9^/L	6.20 ± 1.31	6.71 ± 1.66	-1.410	0.163
PLT, ×10^9^/L	146.00 (135.00, 222.50)	150.00 (138.00, 178.00)	0.000	1.000
HGB, g/L	147.67 ± 11.77	144.60 ± 17.14	0.848	0.399
ALT, U/L	31.00 (26.00, 48.00)	26.00 (17.00, 37.50)	-2.139	0.032
AST, U/L	28.00 (23.00, 38.00)	23.00 (19.00, 27.50)	-2.460	0.014
TBil, µmol/L	11.00 (8.90, 14.80)	11.70 (8.60, 14.60)	-0.270	0.787
Change during treatment				
HBsAg decline at week12, log_10_ IU/mL	-0.42 (-0.86, -0.10)	-0.22 (-0.38, -0.03)	-1.617	0.106
HBsAg decline at week24, log_10_ IU/mL	-0.94 (-2.00, -0.25)	-0.52 (-1.37, -0.28)	-1.007	0.314
ALT elevation from baseline to week 12	34.00 (14.00, 100.50)	21.00 (9.00, 43.50)	-1.778	0.075

Abbreviations: HBeAg, hepatitis B virus e antigen; HBsAg, hepatitis B surface antigen; WBC, white blood cell; PLT, platelet; HGB, hemoglobin; ALT, alanine aminotransferase; AST, aspartate aminotransferase; TBIL, total bilirubin

### Adverse events

Among the 77 patients enrolled in this study, the most commonly reported adverse effects during treatment with PEG-IFN α-2b and TMF were fever (64.9%, 50/77), fatigue (51.9%, 40/77), rash (19.5%, 15/77), and alopecia (19.5%, 15/77). Most AEs were graded as CTCAE grade 1 or 2. Hematologic toxicities were common: neutropenia occurred in 55 patients (71.4%), with 35.1% (27/77) classified as grade 1/2 and 36.4% (28/77) as grade 3. Neutropenia was ameliorated following the administration of leukocyte-elevating agents. Thrombocytopenia was reported in 47 patients (61.0%), including 38.9% (30/77) with grade 1 and 22.1% (17/77) with grade 2 events. All four cases of anemia (5.2%) were grade 2. Elevated ALT and AST levels were observed in 34 (44.2%) and 24 patients (31.2%), respectively. According to CTCAE criteria, ALT elevations were predominantly grade 1, except for four grade 2 and one grade 3 cases; AST elevations were mostly grade 1, with two grade 2 and one grade 3 exceptions. Elevated ALT/AST levels were successfully managed with hepatoprotective drugs, and none of these cases necessitated treatment discontinuation. Neuropsychiatric events were also documented, including insomnia (14.3%, 11/77), depression (2.6%, 2/77), and anxiety (3.9%, 3/77)*)*. Five patients discontinued treatment within 24 weeks due to AEs: three developed rash at various time points, one reported pain and alopecia at week 7, and one had persistent low-grade fever at week 9.

## Discussion

NAs are designed to target and inhibit viral reverse transcriptase, thereby suppressing HBV DNA replication. In contrast, PEG-IFN α exerts its effects via immunomodulatory pathways, enhancing cytotoxic T-cell responses and facilitating the immune-mediated clearance of HBV-infected hepatocytes. Despite its potential, PEG-IFN α is often associated with poor patient tolerance, and its clinical efficacy has not been fully satisfactory [16–18]. Owing to the distinct mechanisms by which PEG-IFN α and NAs exert their effects, combining these therapies could represent a promising strategy to improve treatment outcomes for CHB patients. This study is the first to examine the combination of PEG-IFN α-2b and TMF in CHB patients. Four major findings emerged from our analysis. First, when administered for more than 24 weeks, the combination therapy led to a significant reduction in HBsAg levels, with further decreases observed at both week 36 and week 48. Second, patients in the SR group presented consistently lower HBsAg levels than did those in the NSR group, both at baseline and during treatment. These findings highlight the potential for baseline HBsAg levels to serve as an early indicator of treatment response. The study also revealed that lower baseline HBsAg levels and a more pronounced reduction in HBsAg by week 24 were independent predictors of HBsAg loss. Third, a model integrating baseline HBsAg and its reduction through week 24 provided excellent prediction of serological response (AUC: 0.908), supporting the combined use of static and dynamic viral markers to optimize outcome forecasting. Fourth, ALT levels increased significantly from baseline in treatment-naïve and treatment-experienced patients at week 12, with the TN group showing a more pronounced increase than the TE group. Overall, the combination of PEG-IFN α-2b and TMF had a significant effect on HBsAg loss in CHB patients. Furthermore, baseline HBsAg levels and the degree of HBsAg reduction at week 24 were strong predictors of HBsAg clearance. These findings underscore the potential utility of this combination therapy and highlight the importance of early markers for identifying patients who are most likely to benefit from this treatment approach.

HBsAg loss is considered the most reliable marker of HBV eradication, although complete eradication remains a rare achievement in clinical practice [16, 17]. The loss of HBsAg is associated with a significant reduction in the incidence of liver cirrhosis and HCC, making it the primary treatment goal for CHB patients [16, 17, 19–21]. According to the 2022 Chinese Guidelines for the Prevention and Treatment of Chronic Hepatitis B, TMF has been included as a first-line antiviral treatment option [15]. However, the potential synergistic effect of combining TMF with interferon-based therapies, particularly regarding its impact on HBsAg clearance, remains insufficiently studied. PEG-IFN α-2a and PEG-IFN α-2b are derived from different interferon subtypes, each incorporating distinct pegylation strategies, including variations in the size of the PEG molecule. These differences result in unique pharmacokinetic profiles and dosing regimens for each formulation. Despite these differences, both PEG-IFN α-2a and PEG-IFN α-2b share common therapeutic indications and an identical core mechanism of action, which encompasses direct antiviral effects, immunomodulatory properties, and anti-proliferative activities. Several studies underscore the potential benefits of combining PEG-IFNα with other antiviral agents to enhance HBV suppression and improve serological outcomes. This is exemplified by the ARES study, in which HBeAg-positive CHB patients receiving ETV plus PEG-IFN α-2a add-on therapy demonstrated significantly greater declines in HBsAg, HBeAg, and HBV DNA levels from week 24 to 72 compared to those on ETV monotherapy (all *P* < 0.001) [22]. Marcellin P et al. reported that the highest rate of HBsAg loss (9.1%) was achieved with 72 weeks of TDF plus PEG-IFN α-2a therapy. This outcome was superior to 48-week PEG-IFN monotherapy (2.8%), and 120-week TDF monotherapy (0%) [23]. Li et al. observed mean reductions of 0.65, 0.73 and 1.1 log_10_ IU/mL in HBsAg levels in patients treated with TDF alone, PEG-IFNα add-on, and de novo combination therapy over a 48-week period [24]. In our study, we enrolled both HBeAg-negative and HBeAg-positive patients to evaluate the effects of combination therapy with PEG-IFN α-2b and TMF.

In our study, the median HBsAg level decreased by 2.49 log_10_ IU/mL at week 48. This reduction substantially exceeds that reported by Xiamen Traditional Chinese Medicine Hospital for PEG-IFN α-2b monotherapy (0.91 log_10_ IU/mL) and is superior to that of PEG-IFN α-2b combined with ETV or TDF (1.99 and 1.97 log_10_ IU/mL) [25]. Further analysis by Lin et al. in HBeAg-positive CHB patients revealed that the mean change in HBsAg level in the PEG-IFN α-2b + TDF group was significantly greater than that in the PEG-IFN α-2b + ETV group (1.799 ± 0.3063 vs. 1.078 ± 0.2028, *P* = 0.0491) over the 48-week treatment period [26]. Our study demonstrates that the TMF and PEG-IFN α-2b combination achieves a high virological response rate (94.7% with HBV DNA < 50 IU/mL), which is comparable to the 95–100% rates reported for TDF-based regimens [25, 26]. This collective evidence suggests that tenofovir formulations (TMF/TDF) in combination with PEG-IFN may provide superior virological efficacy compared to ETV-based strategies (78.95-80%) [25, 26]. In HBeAg-positive patients, our TMF and PEG-IFN α-2b regimen facilitated HBeAg clearance in 21.4% of patients by week 48. This efficacy profile is consistent with the 20.45% rate observed in a comparable TDF-based combination regimen [24], reinforcing the role of tenofovir formulations in this combination strategy. Although another study reported a higher rate (40%) for a TDF add-on approach [26], differences in study populations and design preclude a direct comparison. In our study, 26.0% of patients achieved HBsAg loss, and 80% of those developed anti-HBs positivity at the final follow-up. HBeAg-negative patients achieved higher HBsAg clearance (30.2%) compared to HBeAg-positive patients (7.1%, *P* = 0.076). Our findings are in line with those from the “OASIS” project, a large-scale study of 868 CHB patients that demonstrated a 21.5% HBsAg clearance rate after 5 years of PEG-IFN α-2b-based therapy [27]. Similarly, data from Xiamen Traditional Chinese Medicine Hospital showed 48-week clearance rates of 22.03% with TDF combination therapy [25]. Notably, in a specialized cohort of inactive HBsAg carriers, PEG-IFN α-2b plus TDF achieved remarkable clearance rates reaching 55.6% by week 48 [28], highlighting the exceptional potential of combination regimens in selected patient populations. Current clinical guidelines support expanding the indications for hepatitis B antiviral therapy. By extending treatment to a broader patient population, the risk of progression to cirrhosis and HCC can be reduced.

In our study, baseline HBsAg levels and HBeAg positivity rates were significantly lower in the SR group than in the NSR group (all *P* < 0.001). Throughout therapy, the HBsAg levels in the SR group remained consistently lower than those in the NSR group at all measured timepoints (all *P* < 0.001). More importantly, the SR group exhibited significant on-treatment HBsAg declines as early as week 12, with further reduction by week 24 (all *P* < 0.001), highlighting a dynamic response pattern associated with eventual functional cure. Our multivariate regression analysis identified lower baseline HBsAg and greater HBsAg decline at week 24 as independent predictors of HBsAg loss. These findings are strongly supported by a multicenter study led by Hou Jinlin, which identified low qHBsAg levels (HR: 0.293) and HBeAg-negative status (HR: 0.223) as key predictors of HBsAg loss. Additionally, patients achieving HBsAg clearance showed progressive qHBsAg decline during 5-year follow-up, whereas non-responders maintained stable HBsAg levels [29]. The combined model integrating baseline HBsAg and its decline at week 24 achieved an AUC of 0.908. Although not statistically superior to either predictor alone (both *P* >0.05) likely due to the limited sample size, this model provides enhanced clinical utility by capturing both static and dynamic aspects of treatment response, thereby helping to identify patients with a consistently favorable profile across treatment stages. Patients with baseline HBsAg < 2.765 log_10_ IU/mL who achieve an HBsAg decline >0.595 log_10_ IU/mL at week 24 are identified as optimal candidates for continuing the current regimen, as they exhibit a high probability of HBsAg clearance. Conversely, a “no-go” signal should be considered for patients failing to meet these thresholds, particularly those with a week 24 HBsAg decline of ≤ 0.595 log10 IU/mL, indicating a low likelihood of response and warranting re-assessment of the therapeutic strategy. In addition, Hou Jinlin proposed the GOLDEN model, which integrates serial quantitative HBsAg (qHBsAg) measurements throughout follow-up. The model demonstrated superior predictive performance, with AUC values of 0.981 and 0.979 in the training and external validation cohorts, respectively, significantly exceeding the predictive accuracy of a single qHBsAg measurement (0.845 and 0.901) [29]. As the number of subsequent cases increases, we will conduct further research to explore the prediction of HBsAg clearance with interferon combined with TMF treatment and validate the clinical applicability of the GOLDEN model in this context. A study from Sun Yat-Sen University involving 292 patients receiving PEG-IFN α-2b add-on therapy found that baseline age and HBsAg levels were negative predictors of HBsAg loss at week 48, with AUCs of 0.807 and 0.888 for baseline and week 12 HBsAg, respectively [30]. These findings confirm that both baseline HBsAg levels and the magnitude of HBsAg decease during treatment are critical predictors of HBsAg clearance at week 48 of therapy. Importantly, this predictive value extends to patients receiving interferon combined with TMF treatment, reinforcing the utility of HBsAg monitoring in guiding therapeutic decisions. Beyond optimizing existing regimens, achieving a functional cure for CHB will require exploration of novel immunotherapeutic mechanisms. In this context, emerging approaches such as highly durable circular RNA (circRNA) vaccines represent a promising direction for future prophylactic and therapeutic interventions against HBV [31]. The persistent challenge of viral hepatitis worldwide is further underscored by the recent emergence of severe acute hepatitis of unknown origin in children—a condition with elusive etiology and substantial implications for global health systems [32].

This study reported comparable HBsAg clearance rates between TN (25%) and TE (26.7%) patients. In contrast, the Sun Yat-sen University study showed cumulative clearance rates of 21.3% vs. 14.6%, 28.4% vs. 21.0%, and 34.3% vs. 27.8% at weeks 24, 36, and 48 with PEG-IFN α-2b plus NAs therapy in interferon-experienced versus interferon-naïve patients, respectively (all *P* >0.05) [30]. These results suggest that interferon-NA combination therapy leads to similar HBsAg clearance rates regardless of prior NA or interferon exposure, although confirmation in larger cohorts is needed. The median ALT level at week 12 was significantly greater in TN patients than in TE patients (*P* = 0.002). In TN patients, the immune system remains in a prolonged state of immune tolerance, allowing a substantial reservoir of HBV-infected hepatocytes to persist. Interferon therapy activates the JAK-STAT signaling pathway, significantly enhancing the cytotoxic activity of CD8⁺ T cells and NK cells [33]. This immune potentiation facilitates the targeted elimination of infected hepatocytes. Additionally, interferon induces the OAS-RNaseL pathway within hepatocytes, activating apoptosis-related molecules, which triggers hepatocyte death [34]. However, the immunostimulatory effects of interferon are attenuated in TE patients, likely due to prior antiviral exposure, which may dampen the immune response.

Despite the significant findings, several limitations must be considered. First, the relatively small sample size and short follow-up duration of this study may introduce potential biases, limiting the generalizability of the results. To validate and extend these findings, we are actively collaborating with the First Affiliated Hospital of Anhui Medical University and Anhui Provincial Hospital in a multi-center effort to expand the cohort. This ongoing work will enable a more robust and definitive assessment of the treatment’s efficacy. Second, the absence of long-term follow-up data prevents a comprehensive understanding of the sustained therapeutic response to PEG-IFN α-2b and TMF combination therapy. Additionally, the limited number of HBeAg-positive patients precluded a detailed subgroup analysis and increased the risk of type II error. Finally, the impact of HBV genotype on the durability of HBsAg clearance remains unexplored and warrants further investigation.

## Conclusions

This study demonstrated that PEG-IFN α-2b and TMF combination therapy beyond 24 weeks induced significant and progressively greater reductions in HBsAg levels. Lower baseline HBsAg levels and a greater decline in HBsAg from baseline to week 24 are strong predictors of HBsAg loss, and these parameters exhibit high predictive accuracy. Future studies will expand the sample size and compare the therapeutic efficacy of the PEG-IFN α-2b plus TMF regimen against PEG-IFN α-2b combined with TDF or TAF.

## Data Availability

The datasets used and/or analysed during the current study are available from the corresponding author on reasonable request.

## Abbreviations

TMF: Tenofovir amibufenamide
CHB: Chronic hepatitis B
SR: Serologic response
HBsAg: Hepatitis B surface antigen
AEs: Adverse events
NSR: Non-serological response
AUCs: Areas under the curves
HBV: Hepatitis B virus
WHO: World Health Organization
PEG-IFN: Pegylated interferon-alpha
NAs: Nucleos(t)ide analogs
ETV: Entecavir
TDF: Tenofovir disoproxil fumarate
TAF: Tenofovir alafenamide
ISGs: Interferon-stimulated genes
pgRNA: Pregenomic RNA
HBeAg: Hepatitis B e antigen
ALT: Alanine aminotransferase
AST: Aspartate aminotransferase
TBil: Total bilirubin
CTCAE: Common Terminology Criteria for Adverse Events
SE: Standard error
IQR: Interquartile range
OR: Odds ratios
CI: Confidence interval
ROC: Receiver operating characteristic
WBC: White blood cells
PLT: Platelet
HGB: Hemoglobin
Se: Sensitivity
Sp: Specificity
TE: Treatment-experienced
TN: Treatment-naïve

## References

[1] Global Hepatitis Report. 2024: Action for Access in Low- and Middle-Income Countries. 1st ed. Geneva: World Health Organization; 2024.

[2] Wu J, Wang H, Xiang Z, Jiang C, Xu Y, Zhai G, et al. Role of viral hepatitis in pregnancy and its triggering mechanism. J Transl Int Med. 2024;12:344–54. 10.2478/jtim-2024-0015. 39360164 10.2478/jtim-2024-0015 PMC11444475

[3] Yin Y, Chen X, Li X, Hou H, Shi Z. [Intrauterine HBV infection: risk factors and impact of HBV DNA]. Nan Fang Yi Ke Da Xue Xue Bao. 2006;26:1452–4. 17062350

[4] Shi Z. Obstetrical management of fulminant viral hepatitis in late pregnancy. Reprod Sys Sex Disorders. 2012;01. 10.4172/2161-038X.1000102.

[5] Li X-M, Ma L, Yang Y-B, Shi Z-J, Zhou S-S. Prognostic factors of fulminant hepatitis in pregnancy. Chin Med J (Engl). 2005;118:1754–7. 16313765

[6] Yim HJ, Kim JH, Park JY, Yoon EL, Park H, Kwon JH, et al. Comparison of clinical practice guidelines for the management of chronic hepatitis B: when to start, when to change, and when to stop. Clin Mol Hepatol. 2020;26:411–29. 10.3350/cmh.2020.0049. 32854458 10.3350/cmh.2020.0049 PMC7641563

[7] Sadler AJ, Williams BRG. Interferon-inducible antiviral effectors. Nat Rev Immunol. 2008;8:559–68. 10.1038/nri2314. 18575461 10.1038/nri2314 PMC2522268

[8] Wieland SF, Eustaquio A, Whitten-Bauer C, Boyd B, Chisari FV. Interferon prevents formation of replication-competent hepatitis B virus RNA-containing nucleocapsids. Proc Natl Acad Sci USA. 2005;102:9913–7. 10.1073/pnas.0504273102. 15994231 10.1073/pnas.0504273102 PMC1175012

[9] Belloni L, Allweiss L, Guerrieri F, Pediconi N, Volz T, Pollicino T, et al. IFN-α inhibits HBV transcription and replication in cell culture and in humanized mice by targeting the epigenetic regulation of the nuclear CccDNA minichromosome. J Clin Invest. 2012;122:529–37. 10.1172/JCI58847. 22251702 10.1172/JCI58847 PMC3266786

[10] Zhang H, Hu Y, Wu M, Liu J, Zhu X, Li X, et al. Randomised clinical trial: safety, efficacy and pharmacokinetics of HS-10234 versus Tenofovir for the treatment of chronic hepatitis B infection. Aliment Pharmacol Ther. 2021;53:243–52. 10.1111/apt.16196. 33249630 10.1111/apt.16196

[11] Liu J, Wu M, Kai J, Lin M, Zheng Y, Jiang Y, et al. Effect of food on the pharmacokinetics of Tenofovir amibufenamide: A phase I, Randomized, Open-Label, Two-Period crossover trial in healthy adult subjects. DDDT. 2023;17:3061–72. 10.2147/DDDT.S419084. 37840641 10.2147/DDDT.S419084 PMC10572397

[12] Li L, Zhou J, Li Y, Wang F, Zhang D, Wang M, et al. Effectiveness and safety of Tenofovir Amibufenamide and its comparison with Tenofovir Alafenamide in patients with chronic hepatitis B: results from a retrospective real-world study. Front Pharmacol. 2023;14:1165990. 10.3389/fphar.2023.1165990. 37324480 10.3389/fphar.2023.1165990 PMC10267382

[13] Chinese Society of Infectious Disease Chinese Society of Hepatology, Chinese Medical Association. [The expert consensus on clinical cure (functional cure) of chronic hepatitis B]. Zhonghua Gan Zang Bing Za Zhi. 2019;27:594–603. 10.3760/cma.j.issn.1007-3418.2019.08.003. 31594076 10.3760/cma.j.issn.1007-3418.2019.08.003 PMC12769011

[14] Ning Q, Wu D, Wang G-Q, Ren H, Gao Z-L, Hu P, et al. Roadmap to functional cure of chronic hepatitis B: an expert consensus. J Viral Hepatitis. 2019;26:1146–55. 10.1111/jvh.13126. 10.1111/jvh.13126 31087479

[15] You H, Wang F, Li T, Xu X, Sun Y, Nan Y, et al. Guidelines for the prevention and treatment of chronic hepatitis B(version 2022). J Clin Translational Hepatol. 2023:1425–42. 10.14218/JCTH.2023.00320 PMC10500285 37719965

[16] Terrault NA, Lok ASF, McMahon BJ, Chang K, Hwang JP, Jonas MM, et al. Update on prevention, diagnosis, and treatment of chronic hepatitis B: AASLD 2018 hepatitis B guidance. Hepatology. 2018;67:1560–99. 10.1002/hep.29800. 29405329 10.1002/hep.29800 PMC5975958

[17] Lampertico P, Agarwal K, Berg T, Buti M, Janssen HLA, Papatheodoridis G, et al. EASL 2017 clinical practice guidelines on the management of hepatitis B virus infection. J Hepatol. 2017;67:370–98. 10.1016/j.jhep.2017.03.021. 28427875 10.1016/j.jhep.2017.03.021

[18] Sarin SK, Kumar M, Lau GK, Abbas Z, Chan HLY, Chen CJ, et al. Asian-Pacific clinical practice guidelines on the management of hepatitis B: a 2015 update. Hepatol Int. 2016;10:1–98. 10.1007/s12072-015-9675-4. 26563120 10.1007/s12072-015-9675-4 PMC4722087

[19] Anderson RT, Choi HSJ, Lenz O, Peters MG, Janssen HLA, Mishra P, et al. Association between seroclearance of hepatitis B surface antigen and Long-term clinical outcomes of patients with chronic hepatitis B virus infection: systematic review and Meta-analysis. Clin Gastroenterol Hepatol. 2021;19:463–72. 10.1016/j.cgh.2020.05.041. 32473348 10.1016/j.cgh.2020.05.041

[20] Cornberg M, Lok AS-F, Terrault NA, Zoulim F, Berg T, Brunetto MR, et al. Guidance for design and endpoints of clinical trials in chronic hepatitis B - Report from the 2019 EASL-AASLD HBV Treatment Endpoints Conference‡. J Hepatol. 2020;72:539–57. 10.1016/j.jhep.2019.11.003 10.1016/j.jhep.2019.11.003 31730789

[21] Chinese Society of Infectious Diseases, Chinese Medical Association, Chinese Society of Hepatology, Chinese Medical Association. Zhonghua Gan Zang Bing Za Zhi. 2019;27:938–61. 10.3760/cma.j.issn.1007-3418.2019.12.007. [The guidelines of prevention and treatment for chronic hepatitis B (2019 version)]. 10.3760/cma.j.issn.1007-3418.2019.12.007 PMC12813922 31941257

[22] Brouwer WP, Xie Q, Sonneveld MJ, Zhang N, Zhang Q, Tabak F, et al. Adding pegylated interferon to Entecavir for hepatitis B e antigen–positive chronic hepatitis B: A multicenter randomized trial (ARES study). Hepatology. 2015;61:1512–22. 10.1002/hep.27586. 25348661 10.1002/hep.27586

[23] Marcellin P, Ahn SH, Ma X, Caruntu FA, Tak WY, Elkashab M, et al. Combination of Tenofovir disoproxil fumarate and peginterferon α-2a increases loss of hepatitis B surface antigen in patients with chronic hepatitis B. Gastroenterology. 2016;150:134–e14410. 10.1053/j.gastro.2015.09.043. 26453773 10.1053/j.gastro.2015.09.043

[24] Li J, Qu L, Sun X, Liu Y, Gong Q, Yu D, et al. Peg-interferon alpha add‐on Tenofovir disoproxil fumarate achieved more HBsAg loss in HBeAg‐positive chronic hepatitis B naïve patients. J Viral Hepatitis. 2021;28:1381–91. 10.1111/jvh.13571. 10.1111/jvh.13571 34228855

[25] Liang H, Zheng X, Mao Q, Yang J, Ruan Q, Wu C, et al. Comparative efficacy and safety of pegylated interferon-alpha monotherapy vs combination therapies with Entecavir or Tenofovir in chronic hepatitis B patients. Microbiol Spectr. 2025;13. 10.1128/spectrum.02694-24. 10.1128/spectrum.02694-24 PMC12054097 40172187

[26] Lin S, Fu Y, Wu W, Chen T, Chen N, Xun Z, et al. The efficacy of addition of Tenofovir disoproxil fumarate to Peg-IFNα-2b is superior to the addition of Entecavir in hbeag positive CHB patients with a poor response after 12 weeks of Peg-IFNα-2b treatment alone. Int J Med Sci. 2020;17:1458–63. 10.7150/ijms.45658. 32624702 10.7150/ijms.45658 PMC7330670

[27] Tan Z, Kong N, Zhang Q, Gao X, Shang J, Geng J, et al. Predictive model for HBsAg clearance rate in chronic hepatitis B patients treated with pegylated interferon α-2b for 48 weeks. Hepatol Int. 2025;19:358–67. 10.1007/s12072-024-10764-5. 39702655 10.1007/s12072-024-10764-5 PMC12003487

[28] Chen X-B, Liu F-F, Shu F-L, Liu J-Y, Yang J-H. Peginterferon alfa-2b combined with Tenofovir disoproxil fumarate induced high clinical cure rate in inactive chronic hepatitis B virus carriers. Clin Res Hepatol Gastroenterol. 2021;45:101723. 10.1016/j.clinre.2021.101723. 34091082 10.1016/j.clinre.2021.101723

[29] Fan R, Zhao S, Niu J, Ma H, Xie Q, Yang S, et al. High accuracy model for HBsAg loss based on longitudinal trajectories of serum qHBsAg throughout long-term antiviral therapy. Gut. 2024;73:1725–36. 10.1136/gutjnl-2024-332182. 38902029 10.1136/gutjnl-2024-332182

[30] Yang X, Zhang K, Xu Q, Shu X, Mo Z, Xie D, et al. Interferon add-on therapy increased clinical cure significantly for interferon-experienced chronic hepatitis B patients with low HBsAg. Front Immunol. 2022;13:997608. 10.3389/fimmu.2022.997608. 36148219 10.3389/fimmu.2022.997608 PMC9485616

[31] Xie J, Ye F, Deng X, Tang Y, Liang J-Y, Huang X, et al. Circular RNA: A promising new star of vaccine. J Transl Int Med. 2023;11:372–81. 10.2478/jtim-2023-0122. 38130633 10.2478/jtim-2023-0122 PMC10732498

[32] Fu H-J, Zhou M, Huang Z-H, Chen Y-X, Wu X-X. Severe acute hepatitis of unknown origin in children: clinical issues of concern. J Transl Int Med. 2023;11:19–23. 10.2478/jtim-2023-0010. 37533848 10.2478/jtim-2023-0010 PMC10393056

[33] Hu X, Li J, Fu M, Zhao X, Wang W. The JAK/STAT signaling pathway: from bench to clinic. Sig Transduct Target Ther. 2021;6:402. 10.1038/s41392-021-00791-1. 10.1038/s41392-021-00791-1 PMC8617206 34824210

[34] Justesen J, Hartmann R, Kjeldgaard NO. Gene structure and function of the 2’-5’-oligoadenylate synthetase family. Cell Mol Life Sci. 2000;57:1593–612. 10.1007/pl00000644. 11092454 10.1007/PL00000644 PMC11146851

